# Antigen-specific T lymphocyte responses elicited by a DNA – MVA HIV CN54gp140 immunization regime are significantly altered by the TLR4 adjuvant GLA

**DOI:** 10.1186/1742-4690-9-S2-O65

**Published:** 2012-09-13

**Authors:** PF McKay, AV Cope, J Swales, S Joseph, M Esteban, R Tatoud, D Carter, J Weber, RJ Shattock

**Affiliations:** 1 Imperial College, London, UK; 2 MRC Clinical Trials Unit, London, UK; 3 Centro Nacional de Biotecnología, CSIC, Madrid, Spain; 4 Infectious Disease Research Institute, Seattle, WA, USA

## Background

We assessed the antigen-specific CD4 and CD8 T lymphocyte responses elicited by a unique vaccine antigen matched and single Clade C DNA-poxvirus-protein regimen in mice and determined the immunomodulatory effect of the novel micellar formulation of a synthetic TLR4 ligand, GLA-AF (GLA) administered either sequentially or simultaneously with the MVA and protein.

## Methods

Groups of 10 BALB/c mice were primed with plasmid DNA encoding CN54 env/gag-pol-nef and then boosted with MVA-C (env-gag-pol-nef) and CN54gp140 protein with or without GLA-AF. The MVA and protein were either given sequentially at 3 weekly intervals or simultaneously in different legs at 3 and 6 weeks. Cellular responses were assessed at necropsy three weeks after the final immunization. Splenocytes were harvested and analysed for antigen-specific T cell responses using peptide pools spanning the Env and Gag proteins by intracellular cytokine staining and CFSE labelling. Multiparametric flow profiles were analysed using FlowJo and data sets were organized and charted using the SPICE software.

## Results

GLA adjuvanted CN54gp140 substantially influenced the antigen-specific T lymphocyte cytokine expression profiles and proliferative responses in animals primed with DNA and boosted by MVA or those immunized with MVA alone. We observed adjuvant-induced changes in the polyfunctional IFN-γ, TNF-α and IL-2 expression profiles of both the CD4 and CD8 T lymphocyte populations and differential responses to separate peptide pools.

## Conclusion

We have shown that the GLA adjuvant alters both the degree and the nature of the antigen-specific T lymphocyte responses and that these immunomodulatory changes are critically dependent upon the timing of application. These effects are likely due to either the presence of systemic GLA providing a general immunostimulatory environment or enhanced T cell priming in the local draining lymph nodes. This ability to tailor CN54gp140-specific T cell immunity is a valuable tool to be exploited in vaccine design.

